# Antiplasmodial Activity and Safety Evaluation of Three Widely Distributed Polyherbal Antimalarials in Ghana

**DOI:** 10.1155/jotm/8959836

**Published:** 2026-09-21

**Authors:** Lydia Serwaa Gyimah, Godfred Ansah Asiamah, Aaron Opoku Antwi, Eugene Agyei Aboagye, Felix Kwame Zoiku, Evelyn Asante-Kwatia, Arnold Donkor Forkuo

**Affiliations:** ^1^ Department of Pharmacology, Faculty of Pharmacy and Pharmaceutical Sciences, College of Health Sciences, Kwame Nkrumah University of Science and Technology, Kumasi, Ghana, knust.edu.gh; ^2^ Department of Histopathology, Manhyia District Hospital, Kumasi, Ghana; ^3^ Department of Epidemiology, Noguchi Memorial Institute for Medical Research, University of Ghana, Accra, Ghana, ug.edu.gh; ^4^ Department of Pharmacognosy, Faculty of Pharmacy and Pharmaceutical Sciences, College of Health Sciences, Kwame Nkrumah University of Science and Technology, Kumasi, Ghana, knust.edu.gh

**Keywords:** antimalarial, herbal products, malaria, plasmodium, toxicity

## Abstract

Finished herbal antimalarials are widely used in Ghana, although scientific reports regarding their antiplasmodial activity and safety of these products are limited. This study, therefore, assessed the antiplasmodial activities and safety of three widely distributed antimalarial polyherbal products. These products (samples A, B and C) were obtained through a minisurvey conducted in the Accra and Kumasi Metropolises. The SYBR Green fluorescence antiplasmodial assay was conducted on different stages of chloroquine‐resistant and chloroquine‐sensitive strains of *P. falciparum.* Cytotoxicity and subacute toxicity were assessed following MTT assay and OECD guidelines, respectively. Samples A, B and C yielded IC_50_ (μg/mL) values of 0.869 ± 0.10, 0.828 ± 0.05 and 3.07 ± 0.098, respectively, against the chloroquine‐sensitive (3D7) strain at the trophozoite stage. At the schizont stage, samples A, B and C exhibited moderate to weak activities with IC_50_ (μg/mL) values of 9.96 ± 0.63, 7.71 ± 1.63 and 13.66 ± 1.91, respectively. At the gametocyte stage, samples A, B and C displayed moderate to weak activity with IC_50_ (μg/mL) values of 13.59 ± 0.49, 17.92 ± 0.814 and 59.65 ± 1.61, respectively. Parasite inhibition against the chloroquine‐resistant strain (Dd2) at the trophozoite stage demonstrated good antiplasmodial activities for samples A and B, with IC_50_ (μg/mL) values of 0.939 ± 0.04 and 0.318 ± 0.01, respectively. Sample C yielded an IC_50_ (μg/mL) value of 15.09 ± 0.319. In conclusion, the three products are antiplasmodial agents and are safe in the rodent model.

## 1. Introduction

Malaria remains a devastating disease globally. In humans, it is caused by Plasmodium species, including *P. falciparum*, *P. ovale*, *P. knowlesi*, *P. vivax* and *P. malariae*, with *P. falciparum* causing the most fatal cases. The scourge of malaria remains devastating in its morbidity and mortality, especially among pregnant women and children under five. According to the WHO, an estimated 249 million malaria cases, which is above the global tally of cases before the COVID‐19 pandemic, were recorded in 2022, indicating an increase of 5 million when compared to the year 2021 [1]. The highest burden of these cases was recorded in the WHO Sub‐Saharan African region. Additionally, 608,000 malaria‐related mortalities were reported globally, and this, coupled with the morbidities, has been associated with climate change, disruptions in malaria prevention, diagnosis, and treatment failure [1, 2].

The WHO recommends combination therapy over monotherapy for malaria, with the immediate choice of treatment for uncomplicated malaria being the Artemisinin combination drugs. However, its use has been impeded in recent times because of challenges including high cost, inaccessibility, and parasite resistance. Thus, indigenes in various cultures employ traditional medicines, largely involving medicinal plants, which have been widely used for therapeutic purposes over the years. This practice is widespread in many developing countries, such as Ghana. Typically, they are combined and prepared into various products and formulations and sold in various markets [3].

In Ghana, there has been an increase in the patronage of herbal products on the market to treat malaria. This is usually because of the preference for herbal medicines and the acclaimed safety of natural products as compared to conventional drugs [4]. The high patronage of these antimalarial herbal medicines in Ghana raises a public health concern relating to their safety. Adverse drug reactions (ADRs) relating to the use of antimalarial herbal medicines in Ghana are underreported due to the complexity of herbal medicines and a lack of knowledge and awareness. As a result, probable toxicity concerns from repeated use of these medicines are not detected, reported or established.

In Ghana, like other countries, registration requirements for polyherbal medicinal products indicated for malaria do not include the determination of the efficacy of the product [5]. The knowledge of the activities of constituent plants (active ingredients) used in formulation, known from literature, becomes the baseline in accepting the proposed indication. Thus, there are limited efficacy reports of commercial herbal products formulated using multiple plants. Patients, therefore, resort to polyherbal preparations at their own risk since these herbal combinations may not even work in treating their diseases.

The increase in illegal mining activities in Ghana has also raised concerns about heavy metal contamination of raw materials used in herbal drug production. Additionally, microbial contamination of raw materials, which is inevitable in harvesting raw materials, needs to be checked for permissible levels for public safety concerns, thus underscoring the relevance of the quality of herbal products, which usually influences the toxicity and effectiveness of the products.

Therefore, this study aims to evaluate the toxicity, antiplasmodial efficacy, heavy metal and microbial contamination of three (3) widely distributed finished herbal antimalarials in the Kumasi and Accra metropolises.

## 2. Materials and Methods

### 2.1. Materials

Drugs and reagents used are chloroquine (Sigma), artesunate (Sigma), amodiaquine (Sigma), DMSO (Sigma), HEPES, L‐glutamine, hypoxanthine, sodium bicarbonate, RPMI 1640 powder (GIBCO, 10.43 g per pack), RPMI 1640 medium, glucose, AlbuMAX, SYBR Green 1, Giemsa stain, ethanol, methanol, immersion oil, EDTA tubes, SST tubes, Triton X‐100, Na_2_HPO_4_, NaCl, KH_2_PO_4_, sorbitol and glycerol.

### 2.2. Survey of Widely Distributed Antimalarial Products

#### 2.2.1. Population and Study Area

**FIGURE 1:**
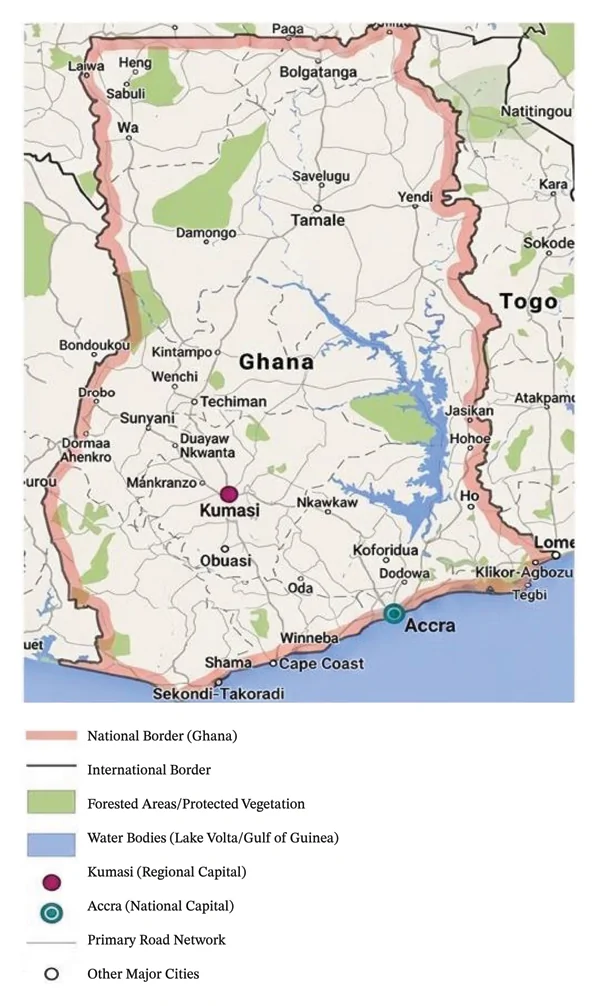
Map of Ghana showing Kumasi and Accra metropolises (Courtesy: Google Maps).

This study employed a survey that was conducted at Kumasi and Accra metropolises, the two biggest cities in Ghana (Figure 1). Currently, the metropolises act as significant crossroads in the nation where the majority of the principal highways intersect. Both northern and southern travellers pass through the metropolises on their way to their destinations. The metropolises still play a significant part in the nation’s and the West African subregion’s trade. Many retailers resort to these metropolises because of the regular supply of products and reduced prices to foster economic gains.

#### 2.2.2. Sampling Technique

The nonprobability sampling technique was used to choose research participants. This sampling method depends on the investigator’s judgement rather than chance [6]. Moreover, within the framework of nonprobability sampling, the research employed the purposive sampling method. This approach has the benefit of guaranteeing that the required pertinent data originate from reputable herbal product wholesalers in both metropolises. Additionally, in the effort to address the research gap, which aims to investigate the rate of distribution of herbal products, purposive sampling is regarded as a suitable technique.

#### 2.2.3. Data Collection Instrument

In collecting data, questionnaires featuring both open‐ and closed‐ended questions, as well as interviews, were utilised. Closed‐ended questions, which require respondents to select from a list of responses, are effective for gathering quantitative data and streamlining data analysis. In contrast, open‐ended questions allow respondents to articulate their opinions and remarks without researcher influence. The interviews included questions that contribute to the research and are fundamental for the respondents’ understanding. Before administering the questionnaires, a pre‐test of the questionnaires was conducted.

#### 2.2.4. Pre‐Testing of Questionnaires

The questionnaires were pre‐tested to elicit required responses, content relevance, time and appropriateness, sequencing, wording, quality aspects and training in a suitable process for administering the questionnaire [7]. Questionnaires were pre‐tested in two establishments to help make the necessary modifications to faults identified.

#### 2.2.5. Administration of the Questionnaires

The researcher administered the questionnaires to the distributors of herbal antimalarial products in Kumasi and Accra metropolises. The researcher visited the selected distributors after attaining the prerequisite clearance and consent.

#### 2.2.6. Selected Products and Marketed Uses

Product A is a herbal preparation used to treat malaria in Ghana. It is used to treat uncomplicated malaria over a course of 1 week. It contains *Aloe schweinfurthii*, *Khaya senegalensis*, *Piliostigma thonningii* and *Cassia siamea*. Product B is a polyherbal product containing *Ocimum viride, Vernonia amygdalina, Morinda lucida, Alstonia boonei* and *Carica papaya*. The product is marketed for the treatment of malaria infection. Product C is a herbal formulation that is used to treat malaria and body pains. The product contains *Ananas comosus, Citrus aurantifolia,* and *Azadirachta indica*.

### 2.3. Biological Assays

#### 2.3.1. Subacute Toxicity Studies

Animal experimental techniques were conducted after the study received ethical permission from the Animal Research Ethics Committee, Kwame Nkrumah University of Science and Technology (KNUST 0073). These studies followed OECD guidelines [8] in evaluating the toxicity profile of the products. Briefly, following a random selection process, 50 albino rats (males and females) were placed in 10 groups (1–10), where a group was made up of 5 rats, which were treated daily with the products orally. Allometric scaling/dose conversion was used to convert the doses on product labels to animal equivalent doses. Animal equivalent doses at three different concentrations for Product A, B and C were coded as A1 ⟶ 28.8 mg/kg, A2 ⟶ 47.2 mg/kg, A3 ⟶ 86.1 mg/kg, B1 ⟶ 13.6 mg/kg, B2 ⟶ 24.4 mg/kg, B3 ⟶ 41.68 mg/kg, C1 ⟶ 331.7 mg/kg, C2 ⟶ 553 mg/kg and C3 ⟶ 1037.6 mg/kg respectively. Product A was given to Groups 1, 2 and 3 at doses of 28.8, 86.1 and 47.2 mg/kg. Similarly, Groups 8, 9 and 10 were treated with doses of product B at 41.68, 13.6 and 24.4 mg/kg. Then, product C was given to Groups 5, 4 and 7 at doses of 553, 331.7 and 1037.6 mg/kg (Table S1). Group 6 (negative control) was given distilled water daily. Treatment with products and water was carried out for 2 weeks. Throughout this period, behavioural changes and other toxic indicators were monitored, alongside measurement of the body weight of animals on a weekly basis. Afterwards, 3 rats from each group were euthanised using isoflurane 1%‐2% in the mixture of 33% oxygen and 66% nitrous oxide in an induction chamber. Anaesthesia was confirmed by the absence of pedal withdrawal reflex [9], followed by the manual administration of blunt force trauma to the cranium using the weight‐drop method [9, 10] after fasting overnight [11] by skilled personnel at the animal house. The death of the rats was confirmed by observing for respiration, pulse, corneal reflex, and firm toe pinch response. Heparinised and nonheparinised samples of blood were obtained in duplicates for the biochemistry and haematology tests.

#### 2.3.2. Haematological and Biochemical Analysis

Routine haematological parameters were assessed from samples of blood from animals using a haematology analyser (Sysmex XN 550, Germany). Similarly, clinical biochemistry was assessed using a biochemistry analyser (Vitros Integrated System 5600 analysers, USA).

#### 2.3.3. Gross Macroscopy and Relative Organ Weight Analysis

Vital organs (the kidney and liver) were removed on the 15th day, washed using NaCl (0.9%), and analysed macroscopically to detect morphological abnormalities. An electronic scale was then used to measure the organs. The relative weights of the organs (ROW) were assessed using the following equation:

(1) ROW=weight of organweight of rat×100.

#### 2.3.4. Histopathological Evaluation of Organs

The excised organs (kidneys and livers) from the euthanised animals were stored in formalin (10%) for histological assessment. Slices of tissues were made, ethanol‐dried, and then fixed in paraffin wax. They were then fixed on microscope slides, and haematoxylin and eosin were applied as a stain. The slides were mounted under a microscope (BX51, Olympus) and photomicrographs were taken using a digital camera (Lumenera, Canada).

### 2.4. Antiplasmodial Assay and Cytotoxicity

#### 2.4.1. Plasmodium Species

Chloroquine‐resistant (CQR) DD2 and chloroquine‐sensitive (CQS) 3D7 strains of *P*. *falciparum* were utilised in the study. They were obtained from Noguchi’s Immunology Department, University of Ghana, Accra, Ghana.

#### 2.4.2. In Vitro Drug Assay

##### 2.4.2.1. Preparation of Compounds

A weight of 1 mg of each freeze‐dried product was separately weighed and added to a 1 mL volume of DMSO (0.5%) to create a 1000 μg/mL solution (stock). This solution was vortexed, filtered through a pore filter unit (0.2 μm) and stored until needed. They were then serially diluted till a concentration of 100 μg/mL was achieved. This was similarly diluted to prepare different concentration ranges from 100 to 0.39 μg/mL.

##### 2.4.2.2. Culturing of Parasite and Preparation for Antiplasmodial Activity Against Trophozoite Stage Parasite

The asexual cultures of the test strains were kept in vitro (under conditions of 90% N_2_, 5% CO_2_ and 5% O_2_) in a complete medium that was made up of 10.44 g/L RPMI 1640, 5.94 g/L HEPES, 5 g/L AlbuMAX II, 50 mg/L hypoxanthine, and 2.1 g/L sodium bicarbonate at 37°C. O^+^ red blood cells were used to culture the parasites, which were then kept in an incubator with the media changed daily till the parasitaemia exceeded 5% ring stages. Following this, sorbitol (5%) was added to the culture in order to acquire a synchronised ring stage of the parasites. The proliferation of parasites was checked by preparing slides stained with Giemsa and observing parasitaemia levels with a X100 lens of a light microscope until a 5% parasitaemia level was exceeded. Using uninfected blood, a parasite suspension containing 2% haematocrit and 1% parasitaemia was made, yielding a total volume of 14 mL in a complete culture medium for plating.

##### 2.4.2.3. Culturing of Schizont Stage of Parasite

CQS 3D7 *P. falciparum* strains were employed in assessing the activity of the products on the parasite’s asexual stages (schizonts). The asexual stages were maintained in vitro using a modified approach [12]. Briefly, cultivation of parasites was carried out in O^+^ red cells and incubated while changing the media daily until the parasite level became higher than 5% ring stages. Afterwards, the parasites were further maintained for the parasitaemia to exceed 8%, with a greater percentage being in the schizont stage. Then, using 60% Percoll (30 mL Percoll + 17 mL RPMI + 3 mL 10x PBS), the parasites were synchronised, poured into a tube (Falcon tube, 15 mL) and spun at 600 rpm, all at room temperature. After discarding the supernatant, the pellet underwent two partial RPMI washes. The last pellet was suspended in a full medium in a tube to produce a haemoglobin content of 20%. About 7 mL of 60% Percoll was measured into a 15 mL Falcon tube, and 2.5 mL of the parasite suspension was slowly laid onto the Percoll and later spun at 700 rpm for 10 min. A 15 mL Falcon tube was filled with approximately 7 mL of 60% Percoll, then 2.5 mL of the parasite suspension was gradually added to the Percoll, which was then spun for 10 min at 700 rpm. A separate Falcon tube was used to hold the schizonts, which appeared as a reddish‐brown ring at the top of the Percoll. Before plating with the different compounds, fresh, uninfected red blood cells and 20 mL of complete medium were added to get the haematocrit down to 2%.

##### 2.4.2.4. Gametocyte Stages Culturing

A volume of 200 μL of the parasite culture was transferred into a T‐25 flask containing 5 mL of CPM after commencing gametocyte culture with haemoglobin of 4%. Once parasitaemia exceeded 8%, the culture was then moved into a fresh T‐75 culture flask. The culture was monitored and left to multiply to roughly 8%–10%. This was done by switching up the media and checking the parasite growth every day using microscopy. At the obtained 10% (ring stages being predominant), the culture was synchronised with sorbitol. This led to obtaining just the rings, and they were managed with prepared CPM and 50 mM N‐acetylglucosamine (Day 0). A flow of gas was carefully passed through the T‐25 flask, sealed firmly, and placed back in the incubator. Then, starting from day 1 to day 4, the spent medium was removed without obtaining blood cells. The flask was filled with CPM‐50 mM N‐acetylglucosamine, gassed, and then put back in the incubator. Day 5 involved removing the used media, adding just CPM to the flask, gassing it, and then putting it back in the incubator. Up until day 10, the culture flask was maintained through repeated gassing procedures. The number of gametocytes per 1000–1500 RBCs was used to estimate the proportion of gametocytes. After creating a parasite suspension, the medication was plated on it.

##### 2.4.2.5. Extract/Compound Plating and Assay

A 96‐well plate was filled with approximately 100 μL of each of the prepared sample concentrations in duplicates. Furthermore, 15 ng/mL of artesunate (positive control) was serially diluted and plated beside the compounds. Also, 100 μL of a parasite mixture containing 2% haematocrit and 1% parasitaemia was then added to each treated well from the second well to the 10th. Moreover, as a negative control, a 100 μL mixture of parasites without any treatment was added to the 11th well, and the process was carried out again for the remaining products. After being exposed to a gas mixture consisting of 90% nitrogen, 5% carbon dioxide, and 5% oxygen for 5 minutes, the plates were placed in a modular chamber and incubated for 72 h at 37°C.

##### 2.4.2.6. SYBR Green Fluorescence Assay

The incubated 96‐well plates were collected 72 h following incubation, and each well received 100 μL of lysing buffer containing SYBR Green. The plates were carefully and gently mixed to prevent the formation of bubbles. The FLUOstar OPTIMA fluorometer plate reader with control software version 2.20 was then used to read the plates at wavelengths of 470 nm and 520 nm after they had been incubated in the dark for 30–60 min.

#### 2.4.3. Cytotoxicity Assay

In duplicates, 100 μL of each diluted extract was transferred into wells of a 96‐well microtiter plate, with concentrations ranging from 6.25 μg/mL to 100 μg/mL. Afterwards, the wells were filled with 100 μL of noninfected red blood cells. The plates were then incubated in a humidified incubator with 5% O_2_ and CO_2_ for 72 h at 37°C. The plate was then incubated for 2 h after 20 μL of 7.5 mg/mL MTT solution (in phosphate‐buffered saline) was added to each well. A volume of 150 μL of culture media was taken out of each well and disposed of after incubation. Then, 200 μL of Triton X‐100 in acidified isopropanol was added to each well to dissolve any formazan that was formed. The plates were then left in the dark for 24 h at room temperature before the optical densities of the wells were measured using a plate reader set to 570 nm.

#### 2.4.4. Elemental Analysis

A quantity of 5 mL of the samples was measured and transferred into a Kjeldahl digestion tube. A 1:3 ratio of nitric and hydrochloric acid was applied. After that, the combination was digested at 450°C until the colour of the solution changed to white, signifying that the digestion had finished. After the digestion process was complete, the combination was decanted into a 100 mL volumetric flask, and the solution was topped off to achieve the 100 mL level. An atomic absorption spectrophotometer was then used to read the solutions for the different elements at the designated wavelengths: as at 193.7 nm, Pb at 217.0 nm, Cd at 228.9 nm, Fe at 248.3 nm and Zn at 213.9 nm.

#### 2.4.5. Microbial Load Determination

The pour plate method, as outlined in the British Pharmacopoeia [13] was utilised in the assessment of the microbial load. The total viable bacteria count was made using nutrient agar, whereas moulds and yeasts were cultivated on Sabouraud dextrose agar. For *Salmonella* species, bismuth sulphite was used, while MacConkey agar was used for *E. coli*. Every medium was prepared per the instructions set by the manufacturer.

#### 2.4.6. FTIR Spectroscopy

The FTIR spectra of the samples were obtained from a PerkinElmer (Model 1600) Fourier transform infrared spectrophotometer at a wavelength from 500 to 400/cm.

#### 2.4.7. Phytochemical Screening and HPLC Fingerprint of Products

The products were qualitatively screened for the presence of phytoconstituents following standard methods [14].

The HPLC fingerprints of the products were generated by chromatographic analysis using a Perkin‐Elmer Flexar HPLC system, which included a binary LC pump, LC autosampler, PDA plus detector, and in‐line degasser, with Chromera software facilitating the procedure. A 0.45 μm membrane filter was used to filter a 10 mL volume of the sample after it had been measured. It was then sonicated for 10 min. After that, the samples were transferred into 2.5 mL vials and filtered using a 0.45 μm membrane filter (Thermo Fisher Scientific, USA) before being injected into the HPLC autosampler. For each product, 3 separate injections (20 μL volume) were made. The components were eluted using a mobile phase consisting of 0.05% TFA (A) and methanol (B) in a linear gradient that transitioned from 95% TFA to 95% methanol over 41 min on a 3.9 × 300 mm Phenomenex C18, 5 μm column at a flow rate of 1 mL/min. The wavelength of detection was 225 nm.

### 2.5. Statistical Analysis

The closed‐ended questions in the questionnaire were coded, while the open‐ended questions were pre‐coded and coded as well. The data input and analysis were done using Python 3.11. The data gathered was analysed both qualitatively and quantitatively. After coding the raw data from the questionnaires, statistical tools such as frequencies were generated.

In experiments involving the use of animals, Dunnett’s post hoc multiple comparison test was used after one‐way ANOVA was used to compare the means of treatment groups to those of the negative and normal control groups. A *p* value of less than 0.05 was deemed statistically significant. Data were expressed as means ± standard error of the mean (SEM). All statistical analyses and graphical representations of data were conducted using GraphPad Prism 8 software (San Diego, CA, USA). CC_50_ (cytotoxic concentration 50%) values, which are 50% cytotoxic concentrations of the samples, were determined from dose‐response curves by nonlinear regression analysis using GraphPad Prism Version 9.5.1 software (GraphPad Software, San Diego, CA, USA). Selectivity index (SI): a ratio measuring a drug’s safety and specificity by comparing how toxic a drug is to normal host cells against how well it kills the parasite (parasite inhibition).

Formula: SI = CC_50_ (host cell toxicity)/IC_50_ (parasite inhibition).

## 3. Results

### 3.1. Survey of Widely Distributed Antimalarial Products

#### 3.1.1. Data Collection

The pre‐testing conducted ensured all questions that needed corrections, additions, and/or subtraction were attended to in ensuring a robust survey. Overall, twenty‐two (22) questionnaires were administered to distributors, and forty‐five (45) different antimalarials were surveyed from the respective distributors.

#### 3.1.2. Distribution of Products

The results from the survey show significant variation in the distribution of different herbal antimalarials. THM stands out with the highest distribution volume at approximately 22,400 units (Figure S1). This indicates its strong market presence and suggests it is a preferred choice among users in the Kumasi metropolis. TMH also shows a high distribution volume of around 17,450 units, reflecting its popularity and potential effectiveness. RHM, with a total distribution of 10,480 units, is another major player, suggesting it is well‐received and likely trusted by consumers. T202HM also distributed a total of 7100 units, indicating significant usage and acceptance. SMM also had a lower total distribution of 2200 units compared to others (Figure S1); its inclusion indicates it has a niche market.

#### 3.1.3. Selection of Widely Distributed Products

Following the results of the survey, six (6) herbal antimalarials were selected because of their wide distribution in both metropolises. These were THM, TME, SMM, RHM, AMMH and T202HM (Figure S1) in decreasing order of patronage. From literature, the toxicity, efficacy and heavy metal content of THM, TME and AMMH have been evaluated [15, 16]. This work therefore selected the remaining three products (SMM, RHM and T202HM) for the various assays. The products revealed both conformity and nonconformity to General Labelling Rule 1992 (L.I. 1541), presented in Table S5. The animal equivalent doses of the selected products were obtained by using allometric scaling of the human doses written on the products’ labels [17–19].

### 3.2. Subacute Toxicity Studies

#### 3.2.1. Effect of Products on Bodyweight

All experimental animals recorded a positive change in weight on day 7, except for the group that received A3. The weights, however, reduced on day 14 for all groups, although the difference was not significant. The weight of rats that received A3 and C2 showed a negative change in weight on Day 14. The control group recorded a positive change in weight for both days (Figure S2).

#### 3.2.2. Effect of Products on Relative Organ Weights

The differences observed between the test and control groups were comparable, except for C1, which had a significant (*p* < 0.05) increment in the relative weight of the liver (Figure S3).

#### 3.2.3. Renal Function Test

Results obtained for urea, sodium, potassium, and chloride were comparable between all groups. Additionally, significant differences (*p* < 0.05) were observed between the B2 group and the control group (Control) for creatinine levels. Similarly, there were significant differences between A1, B1, B2, C3 and CG for the BUN/creatinine levels (*p* < 0.05). Intergroup comparisons also revealed a significant difference between B1 and B2 for creatinine levels and B2 and B3 for BUN/creatinine levels (Table 1).

**TABLE 1:** Renal function test of rats in toxicity assessment.

Parameter	Unit	A1	A2	A3	B1	B2	B3	C1	C2	C3	Control
Urea	mmol/L	6.8 ± 0.7	5.2 ± 0.4	5.8 ± 1.0	7.5 ± 0.6	5.8 ± 0.2	4.6 ± 0.2	5.2 ± 0.8	5.2 ± 0.4	7.3 ± 2.3	4.1 ± 0.5
Creatinine	μmol/L	63.0 ± 7.8	64.0 ± 9.0	60.7 ± 6.5	70.0 ± 5.7	46.7 ± 2.4^∗∗^	58.33 ± 3.8	57.00 ± 4.9	62.33 ± 5.2	67.3 ± 12.0	64.7 ± 4.4
BUN/creatinine	Ratio	51.0 ± 5.1^∗∗∗^	39.9 ± 7.2	44.3 ± 3.2	51.1 ± 6.1^∗∗∗^	58.6 ± 1.6^∗∗∗∗^	37.4 ± 4.0	41.7 ± 3.2	39.9 ± 5.3	49.6 ± 8.5^∗∗^	30.4 ± 5.1
Sodium	mmol/L	142.7 ± 1.2	144.3 ± 1.8	142.0 ± 0.6	143.0 ± 2.0	142.0 ± 1.2	142.3 ± 1.3	142.0 ± 1.0	142.7 ± 0.9	141.3 ± 0.3	141.0 ± 1.0
Potassium	mmol/L	4.5 ± 0.2	4.4 ± 0.4	4.4 ± 0.3	4.0 ± 0.0	4.5 ± 0.1	4.8 ± 0.5	4.3 ± 0.4	4.8 ± 0.1	4.2 ± 0.2	4.3 ± 0.1
Chloride	mmol/L	100.3 ± 0.9	103.7 ± 2.3	101.3 ± 1.8	100.3 ± 0.3	100.0 ± 1.2	101.0 ± 1.0	100.3 ± 1.2	100.0 ± 1.2	99.7 ± 1.9	100.0 ± 0.6

*Note:* Values presented as mean ± SEM, *n* = 3. Values are significantly different at ^∗∗^ *p* < 0.01, ^∗∗∗^ *p* < 0.001 and ^∗∗∗∗^ *p* < 0.0001 when compared to the negative control group (Control).

#### 3.2.4. Liver Function Test

Significant differences (*p* < 0.05) between C2 and the control group (CG) were recorded in the levels of aspartate aminotransferase (AST). In terms of alkaline phosphatase (ALP), significant differences (*p* < 0.05) were recorded for B1 and C3. No significant difference was recorded for all other parameters tested. Significant differences were also recorded between C2 and C1 in AST levels. For ALP levels, a similar observation was made between A2 and A3, C1 and C3 and C1 and C2 (Table 2).

**TABLE 2:** Liver function test of rats in toxicity assessment.

Parameter	Unit	A1	A2	A3	B1	B2	B3	C1	C2	C3	Control
AST	U/L	350.5 ± 15.8	341.0 ± 25.9	357.4 ± 33.0	366.4 ± 24.0	362.2 ± 14.4	379.5 ± 31.3	355.0 ± 14.4	474.1 ± 31.7^∗∗∗^	428.9 ± 131.8	344.6 ± 67.6
ALT	U/L	154.1 ± 21.0	124.4 ± 27.5	96.1 ± 26.6	130.6 ± 13.3	121.9 ± 29.3	147.3 ± 30.3	117.6 ± 28.8	139.3 ± 6.0	77.7 ± 6.2	86.1 ± 7.1
ALP	U/L	289.0 ± 75.9	341.2 ± 55.7	232.7 ± 35.6	339.4 ± 30.8^∗∗^	340.2 ± 21.8	314.4 ± 38.2	272.3 ± 17.2	293.3 ± 42.4	163.5 ± 12.9^∗∗∗^	287.6 ± 45.5
GGT	U/L	6.4 ± 0.5	6.3 ± 0.2	7.3 ± 0.9	6.8 ± 0.1	7.3 ± 1.1	5.7 ± 0.2	6.0 ± 0.6	8.6 ± 0.5	14.5 ± 7.5	6.9 ± 0.3
Total protein	g/L	77.2 ± 2.4	78.0 ± 4.9	68.5 ± 1.3	68.7 ± 0.9	66.0 ± 3.0	62.2 ± 0.9	69.6 ± 3.2	82.6 ± 5.2	58.2 ± 5.6	68.5 ± 1.7
Albumin	g/L	33.8 ± 1.9	34.7 ± 3.9	32.1 ± 0.6	32.1 ± 2.5	32.7 ± 1.5	29.8 ± 1.1	31.3 ± 0.9	35.5 ± 0.7	26.9 ± 5.6	23.7 ± 1.4
Globulin	g/L	43.3 ± 2.8	43.3 ± 1.0	36.4 ± 1.8	36.6 ± 3.3	33.4 ± 1.6	32.3 ± 1.5	38.2 ± 3.6	47.1 ± 5.1	31.3 ± 1.6	38.8 ± 2.8
Total bilirubin	μmol/L	4.3 ± 0.2	4.4 ± 0.1	4.3 ± 0.5	3.5 ± 0.3	5.9 ± 1.7	2.5 ± 0.4	3.2 ± 0.5	6.7 ± 1.7	3.5 ± 1.0	2.9 ± 0.7
Direct bilirubin	μmol/L	0.9 ± 0.3	0.5 ± 0.3	1.3 ± 0.2	0.6 ± 0.1	1.8 ± 0.6	1.0 ± 0.1	0.6 ± 0.0	1.5 ± 0.5	0.4 ± 0.2	1.3 ± 0.3
Indirect bilirubin	μmol/L	3.4 ± 0.3	4.1 ± 0.8	3.0 ± 0.7	2.9 ± 0.6	3.0 ± 1.8	1.5 ± 0.9	2.6 ± 0.8	5.2 ± 1.3	3.2 ± 0.7	1.6 ± 0.5

*Note:* Values presented as mean ± SEM, *n* = 3. Values are significantly different at ^∗∗^ *p* < 0.01, ^∗∗∗^ *p* < 0.001 and ^∗∗∗∗^ *p* < 0.0001 when compared to the negative control group (Control).

#### 3.2.5. Haematological Analysis

Significant differences (*p* < 0.05) were recorded for A1, A3, B2, B3, C1 and C3 for the levels of platelets. Results obtained for the other parameters were comparable (Table 3).

**TABLE 3:** Haematological assessment of rats in toxicity assessment.

Parameter	Unit	Test groups									
		A1	A2	A3	B1	B2	B3	C1	C2	C3	Control
WBC	10^3^/μL	14.8 ± 2.2	14.7 ± 0.9	11.6 ± 0.2	20.9 ± 0.5	15.8 ± 1.3	17.0 ± 1.7	12.7 ± 1.2	16.1 ± 3.2	11.7 ± 2.8	16.1 ± 2.1
RBC	10^3^/μL	7.9 ± 0.6	7.6 ± 0.2	7.3 ± 0.5	8.1 ± 0.3	7.9 ± 0.4	7.4 ± 0.4	8.1 ± 0.0	8.3 ± 0.2	7.23 ± 0.4	7.4 ± 0.1
HGB	g/L	14.3 ± 0.8	13.7 ± 0.4	13.8 ± 0.5	14.3 ± 0.6	14.2 ± 0.4	13.1 ± 0.7	14.0 ± 0.2	14.4 ± 0.5	13.3 ± 0.3	13.1 ± 0.2
HCT	%	46.2 ± 3.1	44.6 ± 1.7	43.7 ± 1.3	45.6 ± 1.6	44.8 ± 1.5	42.3 ± 2.4	44.1 ± 0.6	46.7 ± 1.7	42.2 ± 0.6	42.6 ± 0.9
MCV	fL	58.7 ± 1.5	58.4 ± 0.7	60.1 ± 3.2	56.0 ± 0.8	56.9 ± 1.3	57.5 ± 0.5	54.7 ± 0.9	56.1 ± 0.5	58.6 ± 2.1	57.9 ± 1.8
MCH	Pg	18.2 ± 0.6	17.9 ± 0.2	18.9 ± 0.8	17.5 ± 0.2	18.1 ± 0.4	17.7 ± 0.1	17.3 ± 0.2	17.4 ± 0.2	18.4 ± 0.8	17.8 ± 0.5
MCHC	g/dL	31.0 ± 0.3	30.7 ± 0.4	31.5 ± 0.4	31.3 ± 0.2	31.8 ± 0.2	30.9 ± 0.3	31.7 ± 0.2	30.9 ± 0.2	31.4 ± 0.3	30.8 ± 0.2
PLT	10^3^/μL	922.3 ± 281.3^∗∗∗∗^	850.3 ± 78.8	479.7 ± 158.5^∗∗∗∗^	716.0 ± 54.4	591.7 ± 66.3^∗∗∗∗^	632.7 ± 42.7^∗∗^	656.3 ± 140.9^∗^	823.7 ± 51.7	564.0 ± 190.6^∗∗∗∗^	758 ± 110.3
RDW‐CV	%	18.8 ± 0.4	18.2 ± 1.3	16.8 ± 1.5	17.8 ± 0.5	18.6 ± 0.1	18.7 ± 0.7	18.1 ± 0.5	19.2 ± 0.3	19.3 ± 3.2	18.5 ± 1.1

*Note:* Values presented as mean ± SEM, *n* = 3. Values are significantly different at ^∗^ *p* < 0.05, ^∗∗^ *p* < 0.01 and ^∗∗∗∗^ *p* < 0.0001 when compared to the negative control group (Control).

#### 3.2.6. Histopathology

##### 3.2.6.1. Liver

**FIGURE 2:**
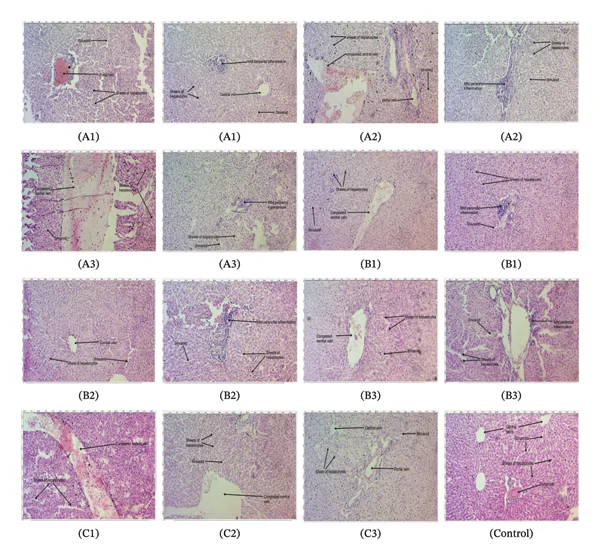
Histopathological micrographs of the liver of rats administered with the three concentrations of product A, B and C. Stain: haemtoxylin and eosin. Magnification: x100. Scale: 2 units equivalent to 1 μm. Periportal chronic inflammation was observed in rats administered with A1, A2, A3, B1, B2 and B3 when compared with control. Congestion (engorged veins) was also observed in the liver of rats treated with A2, A3, B1, B3, C1 and C2 when compared with control, with C3 showing normal liver tissue.

For the control group, sections of liver tissues were histologically normal. No evidence of liver injury, inflammation, degeneration, or malignancy was seen. Periportal chronic inflammation of the hepatocytes was observed in rats administered A1, A2, A3, B1, B2 and B3. Blood vessel congestion was also observed in the liver of rats treated with A2, A3, B1, B3, C1 and C2 (Figure 2) (Table 4).

**TABLE 4:** Lesion grading for congestion and inflammation observed in liver tissues.

Treatment group	Congestion	Inflammation
A1	−	+
A2	+	+
A3	+	+
B1	+	+
B2	−	+
B3	+	+
C1	+++	−
C2	+	−
C3	−	−
Control	−	−

*Note:* + for mild, ++ for moderate, +++ for severe and − for absence.

##### 3.2.6.2. Kidney

Sections of the kidneys were histologically normal in the control group. No evidence of injury, inflammation, degenerative change, or malignancy was observed.

**FIGURE 3:**
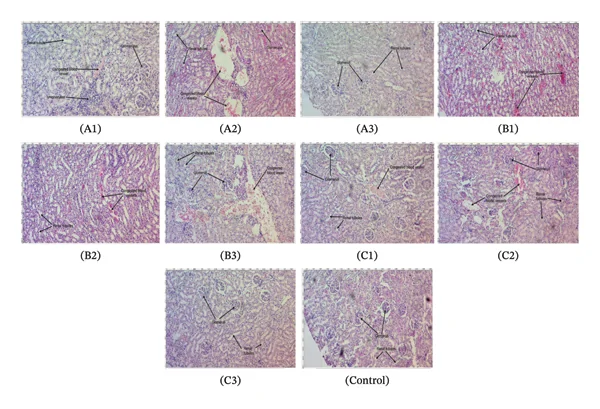
Histopathological micrographs of the kidney of rats administered with the three concentrations of product A, B and C. Stain: haematoxylin and eosin. Magnification: x100. Scale: 2 units equivalent to 1 μm. No abnormalities were seen on the renal cells of rats treated with A3 and C3. Blood vessel congestion was observed in groups treated with A1, A2, B1, B2, B3, C1 and C2. Mild chronic inflammation was also observed in rats treated with A1.

No abnormalities were seen in the renal cells of rats treated with A3 and C3. Engorged veins (congestion) were observed in groups treated with A1, A2, B1, B2, B3, C1 and C2. Mild chronic inflammation was also observed in rats treated with A1 (Figure 3) (Table 5).

**TABLE 5:** Lesion grading for congestion and inflammation observed in kidney tissues.

Treatment group	Congestion	Inflammation
A1	+++	+
A2	++	−
A3	−	−
B1	++	−
B2	++	−
B3	++	−
C1	+	−
C2	+	−
C3	−	−
Control	−	−

*Note:* + for mild, ++ for moderate, +++ for severe and − for absence.

### 3.3. Antiplasmodial Assay

#### 3.3.1. Trophozoite Stages

All products showed good antiplasmodial activity against the 3D7 strain. For the Dd2 lab strain, A and B showed good activity while C showed moderate activity with an IC_50_ > 10 (Table 6).

**TABLE 6:** In vitro cytotoxicity and antiplasmodial activity of products against the trophozoite, schizont and gametocyte stages of *P. falciparum* lab strains.

Product	IC_50_/μg/mL 3D7 (trophozoite)	IC_50_/μg/mL Dd2 (trophozoite)	CC_50_ (μg/mL)	IC_50_/μg/mL 3D7 (gametocyte)	IC_50_/μg/mL 3D7 (schizont)	SI
A	0.8686 ± 0.1031	0.9388 ± 0.0432	117.5970	13.59 ± 0.49	9.958 ± 0.6272	> 100
B	0.8280 ± 0.0487	0.3177 ± 0.0121	64.6284	17.92 ± 0.8135	7.707 ± 1.63	78
C	3.066 ± 0.0978	15.09 ± 0.319	55.2255	59.65 ± 1.606	13.66 ± 1.91	18
Positive control (art)	0.000058 ± 0.000058	0.000204 ± 0.000012	88.5214	0.005550 ± 0.00013	0.000389 ± 0.000014	> 100

*Note:* Values are presented as mean ± SEM (*n* = 3 in two independent experiments).

#### 3.3.2. Schizont and Gametocyte Stages (3D7)

A and B showed good activity while C showed moderate activity against the schizonts of the parasite. Against the gametocytes, C showed low activity while the other products showed moderate activity (Table 6).

### 3.4. Elemental Analysis

Heavy metal (Cd, Pb and As) content in products A, B and C were all found within the permissible limits for herbal medicinal products set in the WHO guidelines for assessing the quality of herbal medicines with reference to contaminants and residues (Table S2). Additionally, product A had the highest recorded Fe and Zn content.

### 3.5. Microbial Load

The total aerobic count of all selected products was within acceptable limits for all products. The total yeast and mould count for product A exceeded the permissible limit (Table S3) according to the standards, microbiological quality of herbal medicinal products for oral use, British Pharmacopoeia [20].

### 3.6. FTIR Spectroscopy

Characteristic IR spectra of the products were developed for the quality assessment. The spectra showed various bands at different frequencies, which could be used for comparisons in subsequent extracts for standardisation purposes (Figure S4).

### 3.7. Phytochemical Screening

The different phytochemicals present in the three (3) products have been outlined in Table S4.

### 3.8. HPLC

HPLC fingerprint of A revealed six (6) peaks, with the most prominent peak being eluted at 23.57 min. For B, out of the three (3) main peaks, the most prominent was eluted at 18.19 min, while the most prominent peak in C was eluted at 5.91 min. The prominent peaks may serve as marker compounds for quality analysis of the various products. The fingerprints would also serve as a standard for quality control of the products (Figure S4).

## 4. Discussion

The Food and Drugs Authority (FDA) of Ghana ensures all herbal products commercialised are duly registered once they conform to all labelling, quality, and acute toxicity standards. The FDA of Ghana, as part of its registration requirements, however, does not require herbal medicine manufacturers to present efficacy studies on their antimalarial herbal products before registration. Also, the toxicity studies required during drug registration are acute in rodents. The endemic nature of malaria and the widespread use of the finished herbal antimalarials imply that patients resort to the highly patronised drugs often, necessitating the need for efficacy and other toxicity studies. This work, therefore, evaluated the toxicity, antiplasmodial efficacy and microbial and heavy metal contamination of three (3) widely distributed finished herbal antimalarials in the Kumasi and Accra metropolises.

Commercial distribution of herbal antimalarials usually proceeds from wholesale distributors to retail sellers in markets, herbal shops, and pharmacies, among others. Distributors function as the primary source of the finished herbal antimalarials. The distributors were thus selected for the survey. Accra is the capital and largest city of Ghana. With a population of over 4 million, the greater Accra metropolitan region (GAMA) is the thirteenth‐largest metropolitan region in Africa and is anchored by Accra, the administrative and economic centre of the greater Accra region [21]. One of Ghana’s biggest metropolitan cities is Kumasi, which is located in the Ashanti region. Ghana’s capital, Accra, is the largest city, with Kumasi coming second. It hosts a number of economic centres with many distributors of finished herbal antimalarials. The two cities thus represented a justifiable representation of the distribution of finished antimalarials in Ghana.

From the survey, six (6) products, THM, TME, SMM, RHM, AMMH and T202HM, were widely distributed in decreasing order of patronage. A study by Wilmot et al. [22] revealed THM to be part of the most highly patronised antimalarial products. Its efficacy studies have been evaluated in earlier studies [22, 23]. TME and AMMH have also been previously worked on for their efficacy and toxicity [15]. This study, therefore, selected RHM, SMM and T202HM for the efficacy and toxicity studies. The selected products and their respective animal equivalent doses were coded for the toxicity and efficacy studies.

Although malaria is an acute condition, necessary toxicity studies, such as subacute toxicity studies, which range from 2 to 4 weeks with continuous dosing, would be important in pharmacovigilance of antimalarials. In the subacute toxicity studies, the animal equivalent doses, obtained from calculating the doses written on the labels of the products, were used. Experimental animals were thus treated with the animal equivalent doses for 14 days [17, 18].

There was an increase in weight on day 7 as compared to the weight of animals before treatment with the products. The differences were comparable among the groups. After comparing to the negative control, it was realised that only the group that received A3 demonstrated a significant weight reduction. The weight increment observed among the groups from day 0 to day 7 could be ascribed to the products improving the appetite of the rats, thereby encouraging feeding, resulting in weight gain [24]. Generally, there was a reduction in the weights from day 7 to day 14 across all the groups. Since this effect was observed in the control group as well, it could be ascribed to physiological factors in the rats or housing and climatic conditions provided at the animal house [25]. Relative organ weight measurements provide insights into potential effects on specific organs. Deviations from normal relative organ weights prompt further investigation. The association of liver and kidney with drug metabolism and its excretion prompts the need to investigate the effect of drugs on their integrity. In the relative organ weights assessment, no significant differences were observed between the test groups and the control on the kidneys. For the liver, a significant increase in weight was observed for the group that received the lowest dose of product C (C1) (Figure 2). The histopathology analysis showed severe congestion (engorged veins) in the liver tissue for the group receiving C1. Severe congestion, which is a result of increased blood volume in veins, can result in oedema formation and hepatocyte swelling, which overall increases liver weight. Hence, an increase in liver organ weight could be ascribed to the engorged veins observed in the tissue. Although no significant changes were observed in the levels of liver enzymes (ALT, AST, ALP and GGT), this observed effect could suggest hepatotoxicity [26]. Other toxicological parameters are, however, needed to validate this observation. These changes were, nevertheless, absent in the group that received the higher doses of product C (C2 and C3). No significant difference was observed in the liver tissue between all other test groups and the control group in the relative organ weight.

Renal function test (RFT) measures the levels of different agents in blood that estimate the normal function of the kidneys. The elevation of these substances/markers as compared to a control group could indicate various damages to the kidney tissues and/or their function [27]. From the results, the levels of urea, sodium, potassium, and chloride were not statistically different between test groups and the control (Table 1). The group that received B2, however, showed statistically (*p* < 0.05) decreased levels of creatinine (Table 1). From the histopathological analysis, moderate congestion was observed in the group treated with B2. Renal congestion affects kidney function, causing changes in creatinine levels [28]. However, poor liver function affects creatine production, resulting in low creatinine levels. High creatinine levels often indicate kidney dysfunction, while low levels are usually unrelated to kidney health [28]. Thus, B2 did not demonstrate toxicity on the kidney. Significant differences (*p* < 0.05) were observed in the BUN/creatinine levels in some groups (Table 1). The BUN/creatinine ratio helps assess issues such as dehydration, kidney injury, gut bleeding, and other problems usually related to acute kidney disease [29]. Rats that were administered A1, B1, B2 and C3 had significantly (*p* < 0.05) elevated BUN/creatinine ratio when compared to the control group (Table 1). Histopathological assessment of kidney tissues showed severe congestion (engorged veins) and mild chronic inflammation with A1, moderate congestion with B1, B2 and B3, and no abnormalities with C3 (Figure 3). Congestion (engorged veins), which is the increase of blood volume in veins of the kidney tissue, increases pressure and reduces kidney perfusion [30]. Reduced kidney perfusion is a contributing factor in elevating the BUN/creatinine ratio [31]. The elevated levels with A1, B1, and B2 could be a result of the congestion observed, though this is different for C3. Additionally, lower levels of albumin affect the BUN/creatinine ratio by activating platelets [32]. Although liver function tests did not show a statistical difference for albumin levels (Table 2) when compared to the control group, haematological analysis showed significantly (*p* < 0.05) high and low platelet levels with A1 and B2, respectively (Table 3). This could also be a contributing factor to the elevated levels of BUN/creatinine ratio [33]. The low doses of products A and B showing significant difference with the control group, as opposed to the higher doses, can be partly ascribed to hormesis. Hormesis is a dose‐response relationship in which a substance exhibits toxic or adverse effects at low doses, but has no effects at high doses [34]. This can also be seen in C3, where no observed effect was seen in the histopathology assessment. Thus, the effects of the drugs on the kidneys of animals are nonlinear, changing disproportionately with the dose. That notwithstanding, hormesis should not be misinterpreted as suggesting low doses of the drug as toxic [34].

In the liver function test (LFT), the AST levels were significantly (*p* < 0.05) elevated in C2 when compared to the control group (Table 2). Elevated AST levels can be an indicator of liver diseases such as cirrhosis and hepatitis [35]. Liver damage or dysfunction can cause AST to leak into the bloodstream, resulting in higher‐than‐normal levels. The histopathological analysis indicated congestion (engorged veins) and lymphoplasmacytic infiltrate for the group treated with C2. Although high AST levels have been associated with congestion in some studies [36], there is no direct link between lymphoplasmacytic infiltrate and congestion raising AST levels [37]. The elevation observed therefore warrants the need to do a thorough investigation to determine the link and to confirm or otherwise the incidence of hepatotoxicity of the drug. Groups B1 and C3 recorded significant elevation and reduction in ALP levels, respectively, when compared to the control group (Table 2). Microscopic view showed a normal liver with C3 when compared to the control and mild congestion with B1. Congestion seen in B1, though mild, may be a marker for the elevated level of ALP. Elevated ALP values are indicators of liver damage such as hepatitis, cirrhosis, and liver cancer. Low levels, however, are not associated with liver condition but could result from malnutrition. *Khaya senegalensis*, a component of product A in earlier studies, has been reported to cause an increase in AST, ALT and ALP levels [38]. This was, however, different from the results of this study; there was no increase in the levels of AST, ALT and ALP in the groups treated with the three concentrations of product A.

In the haematological analysis, there was a significant (*p* < 0.05) elevation in the platelet levels of rats treated with A1. A reduction was observed in the levels of the groups treated with A3, B2, B3, C1 and C3. The differences were not in any trend. All other parameters were not significantly different from the control.

Histological sections of the liver revealed different observations among the groups, including congestion and mild chronic inflammation. A few chronic inflammatory cells (lymphocytes) are seen around the portal triad. Drug‐induced liver injury is a known cause of periportal chronic inflammation (PCI). This, however, does not qualify as periportal chronic inflammation since a few lymphocytes around the portal triad are also seen histologically in normal liver tissue. The liver and kidney parameters were also not significantly different from the control at all dose levels. Other toxicological parameters could be evaluated to conclusively validate or otherwise the toxicity of all products.

The reduction in cell viability, which may be reversible or irreversible, depending on the severity, defines cytotoxicity. The CC_50_ is a classification used to measure the cytotoxicity of substances. A CC_50_ > 90 μg/mL is classified as not cytotoxic, CC_50_ between 2 and 89 μg/mL is classified as moderately cytotoxic, while CC_50_ < 2 μg/mL is classified as cytotoxic according to Philippe et al. [39]. Product A had a CC_50_ of 117.6 μg/mL, hence is classified as not cytotoxic, while products B and C are moderately toxic.

The therapeutic activity of medicinal plants is attributed to the secondary metabolites present in them. The secondary metabolites are influenced by the geographical location and environmental factors. The levels of heavy metals present in soils can invariably be taken up by plants and consequently contribute to the efficacy and/or safety of herbal preparations [40]. The levels of all metals tested were within acceptable limits as they conformed to standards. This indicates that raw materials used in the formulation of all products are free from heavy metal poisoning; thus, the products would not raise the concern of heavy metal poisoning. The plant parts were likely sourced from regions with minimal contamination by toxic metals.

Microbial contamination of finished herbal products at all levels was not observed, as all were within standards (microbiological quality of herbal medicinal products for oral use, British Pharmacopoeia) [20], except for product A, exceeding total yeast and mould count. This could result from the raw materials used in the formulation, as well as not adhering to good manufacturing practices. The contamination of plant materials utilised in herbal formulations might compromise final products if appropriate formulation protocols are not adhered to [41].

The antiplasmodial activities of the aqueous extracts were categorised according to the classification by Philippe et al. [39]. All selected products were highly active (IC_50_ < 5 μg/mL) against the trophozoite stage of the 3D7 *P. falciparum*. Products A and B were similarly highly active against the trophozoite stage of Dd2 *P. falciparum,* while product C was weakly active (15 < IC_50_ < 50 μg/mL) (Table 4). All products were also moderately active (5 < IC_50_ < 15 μg/mL) against the schizont stages of 3D7 *P. falciparum.* Product A was similarly moderately active against the gametocyte, product B was weakly active, and product C was inactive (IC_50_ > 50 μg/mL) (Table 4). The products were more active at the trophozoite stages of the parasite as compared to the schizont and gametocyte stages. Thus, the products would be recommended for curative rather than prophylactic or radical cure. This effect can be ascribed to the constituent plant extracts, which are used folklorically and also reported as antimalarial agents [38, 42]. The effects of herbal medicines are attributed to their phytochemicals. From the phytochemical screening (Table S4), the presence of various secondary metabolites was detected in the products. The antimalarial activity of secondary metabolites, including alkaloids [43, 44], triterpenoids [45, 46], saponins [47, 48] and tannins [49, 50], has been reported. These constituents present in the constituent plants may be acting either in isolation or in combination to produce the observed effects.

The extensive use of the products necessitates the establishment of standards for their quality control. Quality control of herbal medicines, involving analytical techniques including chromatographic and spectroscopic methods, has gained much attention in recent years [51]. The upsurge in the use of herbal medicines in the global market has also prompted the valorisation of herbal medicines using these techniques. From the results, the HPLC and FTIR fingerprints obtained can serve as benchmarks for checking adulteration of the products. Also, the microbial load, phytochemical screening and elemental analysis of the products would serve as quality control parameters for further evaluation of the products.

## 5. Conclusion

Three (3) commercial polyherbal products, A, B and C, were selected for safety and efficacy studies following the survey. All selected products were considered safe in terms of the relative organ weights, body weights, haematological effects and heavy metal content. Although for biochemical and histological effects, congestion and inflammation were observed in tissues of some treatment groups, and significant changes in some biochemical parameters in some treatment groups, they do not conclusively indicate hepatotoxicity or kidney failure; further investigations need to be conducted to confirm drug toxicity for all products. All products were considered safe in terms of microbial effects except for product A, having total yeast and mould count exceeding acceptable limits. The products displayed antiplasmodial activities against the trophozoite (3D7, Dd2), schizont and gametocyte (3D7) stages of *Plasmodium falciparum*. HPLC and FTIR spectra were generated for quality control purposes.

## Funding

The authors did not receive any financial support for the research, authorship, and/or publication of this article.

## Ethics Statement

Animal experimental techniques were conducted after the study received ethical permission from the Committee on Animal Ethics and Research, Department of Pharmacology, Kwame Nkrumah University of Science and Technology (KNUST/Cology/042).

## Conflicts of Interest

The authors declare no conflicts of interest.

## Supporting Information

Additional supporting information can be found online in the Supporting Information section.

## Supporting information


**Supporting Information** Table S1: dosing and coding of products. Doses and codes of products used in biological assay were determined from allometric scaling of the human equivalent doses. Codes A1–C3 were used to ensure confidentiality of the products used. Table S2: heavy metal quantities in widely distributed products. The levels of toxic heavy metals, including Cd, As and Pb, were all within permissible limits in products A, B and C. The beneficial elements such as Zn and Fe were in appreciable quantities. Table S3: microbial load of selected antimalarial products. The microbial load, including the total aerobic and total yeast and mould counts, was within allowable levels for products B and C. While the total aerobic counts were within permissible limits for product A, its total yeast and mould counts exceeded levels allowed by the British Pharmacopoeia. Table S4: phytochemical screening of selected products. The preliminary phytochemical screening of all products revealed the presence of numerous phytoconstituents including tannins, saponins, flavonoids, reducing sugars, alkaloids, triterpenoids and coumarins. Phytosterols were not detected in any of the products sampled. Table S5: products conformance to regulatory instrument (L11541). The conformance of the products to L11541 reveals that the products conform to parameters such as active ingredients and the manufacturer’s details, but nonconformity is observed in parameters such as storage conditions, shelf life, dosage, and date markings. Figure S1: distribution of herbal antimalarials in Kumasi and Accra metropolises. The distribution of herbal antimalarial products reveals high quantities for a few drug codes, moderate levels for some, and low distribution for the majority, indicating a skewed distribution pattern. Figure S2: percentage change in weight of rats in subacute toxicity study. The percentage change in body weight across the products shows an overall increase at Day 7, with variations ranging from weight gain to slight weight loss. By Day 14, weight changes are generally reduced, with some products showing minimal gains and others slight decreases. Figure S3: relative organ weights of rats in toxicity test. There were no significant differences (*p* > 0.05) between the relative organ weights of the control and the product‐treated groups. This was, however, not the case for the relative liver weight of the rats treated with C1 and the control group, as there was a significant difference (*p* < 0.05). Figure S4: FTIR and HPLC fingerprints of samples A, B and C. The FTIR spectra and HPLC chromatograms provide fingerprints for quality evaluation of the sampled products. The HPLC chromatogram of product C reveals numerous constituents, which are more than those of products A and B.

## Data Availability

All datasets used and/or analysed during the current study are available in the manuscript.
