# Supplementary material for: Antiplasmodial Activity and Safety Evaluation of Three Widely Distributed Polyherbal Antimalarials in Ghana
Source: J Trop Med. 2026 Sep 21;2026:8959836. doi: 10.1155/jotm/8959836 (PMC13594769; doi:10.1155/jotm/8959836)
Supplement: Supplementary file 1 [file JOTM-2026-8959836-s001.docx]

**SUPPLEMENTARY MATERIALS**

Table S1: Dosing and coding of products

Doses and codes of products used in biological assay, determined from allometric scaling of human equivalent doses.

| Product | Dose (mg/kg) | Code |
| --- | --- | --- |
| Product A | 28.8 | A1 |
|  | 47.2 | A2 |
|  | 86.1 | A3 |
| Product B | 13.6 | B1 |
|  | 24.4 | B2 |
|  | 41.68 | B3 |
| Product C | 331.7 | C1 |
|  | 553 | C2 |
|  | 1037.6 | C3 |

Table S2: Heavy metal quantities in widely distributed products.

The levels of toxic heavy metals, including Cd, As, and Pb, were all within permissible limits in products A, B and C. Beneficial elements such as Zn and Fe were in appreciable quantities (Table S2).

| Heavy Metals | A (mg/L) | B (mg/L) | C C(mg/L) | Reference value (mg/L) |
| --- | --- | --- | --- | --- |
| Fe | 5.113±0.370 | 3.573±0.070 | 4.587±0.272 | NS |
| Cd | 0.014±0.002 | 0.052±0.004 | 0.113±0.044 | 0.3 |
| As | 0.069±0.006 | 0.046±0.002 | 0.034±0.004 | 5 |
| Zn | 6.783±1.022 | 1.507±0.051 | 1.61±0.087 | 50 |
| Pb | 0.051±0.004 | 0.065±0.0004 | 0.035±0.002 | 10 |

Table S3: Microbial load of selected antimalarial products

The microbial load, including the total aerobic and total yeast and mould counts, were within allowable levels for products B and C. While the total aerobic counts were within permissible limits for product A, its total yeast and mould counts exceeded levels allowed by the British Pharmacopoeia.

| Sample | Total aerobic Counts | Total yeast and mould count | *E. coli* | *S. Typhi* |
| --- | --- | --- | --- | --- |
| A1 | 1.93 x 10^2^ | 6.63 x 10^2^ | Nil | Nil |
| B1 | 5.00 x 10^1^ | 9.00 x 10^1^ | Nil | Nil |
| C1 | 2.43 x 10^2^ | 0.67 x 10^1^ | Nil | Nil |
| BP 2023 | ≤ 5.00 x 10^4^ | ≤ 5.00 x 10^2^ | absent | absent |

Table S4: Phytochemical screening of selected products

Preliminary phytochemical screening of all products revealed the presence of numerous phytoconstituents, but phytosterols in all products.

| Phytochemical | A | B | C |
| --- | --- | --- | --- |
| Tannins | + | + | + |
| Saponins | + | + | + |
| Flavonoids | + | + | + |
| Reducing sugars | + | + | + |
| Alkaloids | + | + | + |
| Triterpenoids | + | + | + |
| Phytosterols | - | - | - |
| Coumarins | + | + | + |

Key: + = detected, - = not detected

Table S5: Products conformance to L11541

The conformance of the products to L11541 reveals the products conform to parameters such as active ingredients and manufacturer’s details, and non-conformity to parameters such as storage condition, shelf life, dosage and date markings.

| Samples | Conformance to General Labelling Rule 1992 ( L.I. 1541) | | | | | |
| --- | --- | --- | --- | --- | --- | --- |
|  | Active Ingredients | Manufacturers Details | Storage Condition and Caution Statement | Shelf Life | Dosage | Date Markings |
| Sample A | Conforms | Conforms | Conforms | Does not Conform | Does not include age range | Does not Conform |
| Sample B | Conforms | Conforms | Conforms | Does not Conform | Does not include age range | Does not Conform |
| Sample C | Conforms | Conforms | Conforms | Does not Conform | Does not include age range | Does not Conform |

Figure S1: Distribution of herbal antimalarials in Kumasi and Accra metropolises

Figure S2: Percentage change in weight of rats in sub-acute toxicity study.

Figure S3: Relative organ weights of rats in toxicity test

Figure S4: FTIR and HPLC fingerprints of Samples A, B and C.
